# Altered Rhythmicity, Depressive Ruminative Thinking and Suicidal Ideation as Possible Correlates of an Unrecognized Autism Spectrum in Patients with Borderline Personality Disorder

**DOI:** 10.3390/brainsci14121297

**Published:** 2024-12-23

**Authors:** Ivan Mirko Cremone, Liliana Dell’Osso, Benedetta Nardi, Federico Giovannoni, Francesca Parri, Cristiana Pronestì, Chiara Bonelli, Gabriele Massimetti, Stefano Pini, Barbara Carpita

**Affiliations:** Department of Clinical and Experimental Medicine, University of Pisa, 56126 Pisa, Italy; ivan.cremone@gmail.com (I.M.C.); liliana.dellosso@unipi.it (L.D.); f.giovannoni10@gmail.com (F.G.); francyparri@icloud.com (F.P.); cristianapronesti@gmail.com (C.P.); chiarabonelli.95@hotmail.it (C.B.); gabriele.massimetti@unipi.it (G.M.); stefano.pini@unipi.it (S.P.); barbara.carpita@unipi.it (B.C.)

**Keywords:** borderline personality disorder, autistic traits, affective symptoms, suicidality, circadian rhythmicity

## Abstract

Background/Objectives: Recent research has explored the presence of subthreshold autistic traits (ATs) in individuals with borderline personality disorder (BPD), suggesting that these traits may contribute to the severity of BPD symptoms and increase the risk of other mental health issues, including suicidal behaviors. This study aims to investigate the relationship between ATs and affective symptoms, such as mood instability and suicidality, in people diagnosed with BPD. Methods: A total of 48 subjects with BPD were assessed with self-report questionnaires including the Adult Autism Subthreshold Spectrum (AdAS Spectrum), the mood spectrum self-report version (MOODS-SR) and the ruminative response scale (RRS). Results: Subjects with significant ATs scored higher than BPD subjects in all domains and in the total score of AdAS Spectrum, RRS, and MOODS-SR, as well as in the items investigating suicidality. RRS total score, its depression domain, and the MOODS-SR rhythmicity domain, as well as suicidality, were predictors of the presence of ATs. Conclusions: Our data confirm the relationship between the presence of clinically significant ATs and affective symptoms, ruminative thinking, and suicidality in patients with BPD.

## 1. Introduction

Borderline personality disorder (BPD) is a condition characterized by a pervasive pattern of alteration of interpersonal relationships, unstable mood, and self-image, associated with marked impulsivity, with an onset in early adulthood [1]. The core elements of BPD include fear of abandonment, unstable relationships with hyper-idealization alternating with devaluation, instability in self-esteem, impulsivity and self-harming behaviors, marked mood reactivity, chronic feelings of emptiness, and the presence of paranoid ideation or severe dissociative symptoms [1]. The estimated lifetime prevalence of BPD is around 5.3% [2] and approximately 80% of affected individuals are women [3]. Personality disorders are quite common in daily clinical practice; up to 45% of all outpatients have them in addition to other diagnoses [4] and, due to the therapeutic difficulties it poses, BPD is by far the most studied of them. Moreover, impulsivity and self-injurious behaviors make BPD patients at high risk for suicidal tendencies. Indeed, it has been estimated that people with BPD attempt suicide an average of three times during their lifetime [5], and up to 10% of BPD patients die by suicide [6]. Ultimately, its early onset, widespread prevalence, and suicidal risk make BPD a major burden for public mental health, requiring specific rehabilitation programs and constant monitoring.

Interestingly, in recent times, many researchers have focused on the evaluation of the presence of autistic traits (ATs) in individuals with BPD and on their influence on the psychopathological trajectory and manifestation. The interest on subthreshold ATs has risen thanks to the growing body on evidences that are suggesting how they may both represent a factor of vulnerability for the subsequent development of other psychopathological conditions and shape the course of the illnesses [7,8,9]. The presence and correlates of ATs were firstly studied among first-degree relatives of patients affected by ASD, which reported personality traits similar to those of individuals with ASD but not as marked, such as inflexibility, a tendency to isolate, a detached personality, and strong and narrow interests [10,11]. Later, significant ATs were investigated in both clinical and non-clinical population, and higher levels were found among patients suffering from other mental disorders [12,13,14,15,16,17]. In particular, the reason for the interest in subthreshold autistic features is that they appear to have a negative impact on quality of life and constitute a major risk factor for the emergence of other mental conditions, worsening their course and favoring the development of suicidal thoughts and behaviors [17,18,19,20].

In this framework, several studies have also highlighted how ASD and BPD share some nuclear symptoms [21,22,23]. For instance, intense or instable connections, shallow friendships, and the propensity to act out rather than express feelings, although being prevalent traits of BPD, are also common in ASD. Moreover, self-harming behaviors were also consistently reported by ASD subjects, which is another trait that BPD and ASD share [24]. Similarly, BPD patients also commonly report some features that are nuclear in ASD, such as difficulties in verbal and nonverbal communication, social functioning, incorrect assumptions about others’ intentions, and emotional meltdowns [25,26,27,28]. Additionally, numerous studies have documented that individuals with BPD have trouble recognizing, differentiating, and integrating their emotions with those of others [29]. This is in addition to the fact that many BPD traits may be associated with emotional dysregulation, a dimension that is also reported in ASD subjects [30]. Moreover, BPD is frequently comorbid with mood disorders, and the two conditions share several features, such as mood instability [31]. On the other hand, many authors have highlighted a link between ASD and mood disorders, whose onset may be favored by the presence of ASD or also subthreshold ATs [12]. A growing body of evidence also suggests the existence of a correlation between ASD and circadian rhythm alterations, which are very common in mood disorders [32,33,34].

To date, multiple clinical observations have pointed to a connection between BPD and ASD, particularly among individuals with high-functioning autism [35]. Aside from a pure question of comorbidity, some researchers have proposed that there may be shared underlying causes [36]. In this framework, several studies have highlighted the significant overlap between ATs and BPD, with some researchers proposing that certain autism-related dimensions—such as challenges with communication, empathy, and mentalization—may contribute to the development of BPD and increase vulnerability to the disorder. Similarly, a history of trauma in individuals with ATs—traits that involve challenges in social cognition, limited adaptability, repetitive thinking, and sensory sensitivities—might contribute to the development of a condition resembling “complex post-traumatic stress disorder” (cPTSD), which shows similarities to BPD [19,37]. Additionally, recent findings have explored the relationship between autistic traits and specific symptoms of BPD, shedding light on how these traits may affect the progression of the condition [25,38,39]. For instance, studies have identified a notable connection between elevated autistic traits and heightened suicidal tendencies, as well as greater exposure to physical or sexual abuse in individuals with BPD [39,40]. This link between autism spectrum traits and suicidality in BPD patients aligns with previous researches that highlighted a higher rate of ASD comorbidity among BPD patients who had made five or more suicide attempts compared to those with fewer attempts. In accordance with the current literature, some researchers suggested that both ASD and ATs may contribute to an increased risk of suicidality, including both suicidal thoughts and behaviors, in individuals with BPD, highlighting the need for a timely and precise clinical assessment in order to plan targeted and effective therapeutic interventions.

Interestingly, despite the fact that sleep and circadian rhythm problems are prevalent in BPD [41], they have long received less attention than other disorders, such as mood disorders, for which they are part of the diagnostic criteria, and autism, for which they have been thoroughly studied. Circadian rhythms are 24 h physiological oscillations that underpin a temporal architecture of sleep/wake behavior [42]. The circadian system comprises a network of endogenously oscillating 24 h rhythms, known as circadian rhythms, that operate across multiple physiological and behavioral domains [43]. This system consists of multiple autonomous, self-sustaining oscillators throughout the brain and periphery. Hierarchical control over the circadian network is exerted by the central circadian pacemaker or “master clock” in the suprachiasmatic nuclei of the anterior hypothalamus [44]. The master clock creates a synchronized internal day of the circadian clock by coordinating the phase of peripheral oscillators with a steady phase across the body. The pathophysiology of a number of mental illnesses is linked to disruptions in the circadian rhythm in behavioral and endocrine measurements [45,46,47]. However, even though circadian rhythm sleep disorders patients present a higher prevalence of personality disorders (including BPD) [48], to date, circadian disturbances have been inadequately investigated and, as a result, mistreated in BPD [49]. Even though this area of study is very young, investigations looking into changes in circadian rhythmicity in BPD patients are showing encouraging results [50,51,52]. Specifically, some authors have proposed that disruptions in circadian rest–activity rhythms are linked to dimensional qualities like mood instability and impulsivity, which are also key symptoms of BPD [53,54]. Recent studies have also shown links between the intensity of symptoms, suicidal thoughts, and the non-24 h circadian rhythm period in BPD [55]. Additionally, desynchronization between the diurnal rhythm phases has been proposed as a factor in the progression of the illness state [56]. Additionally, recent studies have demonstrated that individuals with BPD have higher levels of phase delay and rhythm desynchrony than those with bipolar illness, demonstrating a more distinct correlation between mood instability and varied rhythms [57]. These findings further corroborate the prominent role of a disrupted circadian component in BPD. Although this theory has not yet been further developed, it has also been suggested that disrupted circadian rhythm function plays a part in the origin of BPD [58].

In this context, the aim of our study was to investigate the presence and symptomatic correlates of ATs in a sample of patients diagnosed with BPD. In particular, our study focused on the evaluation of the relationship between the presence of ATs and affective symptoms, including mood, energy, and cognition, as well as suicidal thoughts and behaviors. Particular attention was also paid to the evaluation of the relationship between ATs and suicidality, in light of the previously reported data.

## 2. Materials and Methods

### 2.1. Study Sample and Procedures

For the purpose of the study, a sample made of 48 patients with a clinical diagnosis of BPD was recruited among in- and out-patients who received a clinical diagnosis of BPD at the psychiatric clinic of Azienda Ospedaliera Universitaria Pisana. The diagnosis of BPD was made through clinical evaluation by expert psychiatrist and on the basis of a careful analysis of the patient’s medical history and medical records; the diagnosis was then confirmed with the SCID-5-PD. The patients recruited were long-followed patients attending the psychiatric clinic of the University of Pisa. The recruitment process excluded subjects with mental disabilities, under 18 or over 65 years, with language or intellectual impairments that would make it impossible to complete the exams, and with ongoing psychotic symptoms. The presence of mood or anxiety disorders was excluded through the use of the SCID-5-RV and clinical and anamnestic evaluation at the time of recruitment. However, a small number of participants were contemplated to be experiencing a depressive episode or anxiety symptoms as long as the such symptoms were not as severe as those of the BPD.

The local Ethics Committee authorized all hiring and evaluation procedures, and the study was conducted in accordance with the Declaration of Helsinki. After receiving a comprehensive explanation of the study and having the opportunity to ask questions, all participants gave signed informed consent.

All participants were evaluated with the self-report questionnaire Adult Autism Subthreshold Spectrum (AdAS Spectrum), the mood spectrum self-report (MOODS-SR), and the ruminative response scale (RRS).

### 2.2. Measures

#### 2.2.1. The AdAS Spectrum

The AdAS Spectrum is a 160-item self-report questionnaire designed to assess a wide variety of autism-related symptoms in individuals without cognitive or language impairments. The questionnaire is composed of seven domains: childhood and adolescence, verbal communication, non-verbal communication, empathy, inflexibility and adherence to routine, restricted interests and rumination, and hyper- and hypo-reactivity to sensory input. Excellent internal consistency (Kuder–Richardson coefficient = 0.964), outstanding test–retest reliability, and convergent validity with other dimensional measures of the autism spectrum were found in the validation research [59]. The AdAS Spectrum questionnaire has two validated threshold scores: above 70 for the identification of subjects with full-blown ASD, and above 43 for determining the presence of significant autistic traits [60].

To date, the questionnaire has been used to assess the presence of autistic traits in BPD patients in numerous researches [19,25,38,39,61].

#### 2.2.2. The MOODS-SR

The MOODS-SR is an extensive assessment tool designed to assess states of depression, mania, and hypomania. It encompasses symptoms, behaviors, and lifestyle choices linked to different levels of mood dysregulation, embracing both severe and mild affective abnormalities. With 160 items divided into seven domains, it assesses manic mood, manic energy, and manic cognition as well as depressive mood, depressive energy, and depressive cognition. Another domain specifically investigates rhythmicity and vegetative processes, which includes eating, sleep patterns, and sexual behavior. During the validation study, the MOODS-SR showed great internal consistency for all domains (Kuder–Richardson coefficient ranging from 0.79 to 0.92) [62]. For the purpose of our study, the MOODS-SR items 102 to 107 were used to assess the presence of suicidal ideation and suicidal behaviors. To date, items 102–107 of the MOODS-SR have been extensively used for assessing suicidality in clinical and non-clinical samples [39,61,63,64,65].

#### 2.2.3. The RRS

The RRS is a 22-item self-report questionnaire, scored on a 4-point Likert scale, designed to measure the inclination toward ruminative thinking. The questionnaire is divided into three domains: brooding, reflection, and depression, and has demonstrated strong validity and reliability [66]. The coefficient of the internal consistency (Cronbach α) of RRS was 0.92.

### 2.3. Statistical Analysis

We divided the sample in two groups based on the score obtained at the AdAS Spectrum questionnaire. Subjects who scored higher than the threshold score of 43 for the presence of significant autistic traits (AT) were included in the BPD-AT group, while those who scored lower were included in the BPD group. Chi-square and *t*-test were used to compare socio-demographic variables between groups.

Student’s *t*-test was then used to compare AdAS Spectrum domains and total score, MOODS-SR subscale mania and depression scores as well as its total, and RRS domain and total scores among the two diagnostic groups. Further Student’s *t*-tests were then used to compare the scores obtained by the two diagnostic groups in all MOODS-SR domains and in the MOODS-SR suicidal ideation and behaviors.

Subsequently, we performed four logistic regressions. The first was carried out using the presence of clinically relevant ATs as dependent variable and MOODS-SR and RRS total scores as independent variables, in order to evaluate if the questionnaires total scores were statistically predictive of significant ATs. The second and third logistic regressions were performed using the same dependent variable and RRS and MOODS-SR domains as independent variables, respectively, in order to assess which RRS and/or MOODS-SR domains were statistically predictive of significant ATs. Lastly, the fourth logistic regression was performed using the same dependent variable and MOODS-SR suicidality scores as independent variables, in order to evaluate if and which suicidality factor was a statistical predictor of significant ATs.

All statistical analyses were performed with SPSS version 26.0.

## 3. Results

The sample recruited for the study was made up of 48 patients with a clinical diagnosis of BPD. Based on the threshold score of 43 of the AdAS Spectrum questionnaire for clinically relevant ATs, the overall sample was divided in two groups: 19 subjects without significant ATs (BPD group), and 29 with relevant ATs (BPD-AT). Only eight subjects out of forty-eight scored over the AdAS Spectrum threshold of 70 for possible full-blown ASD. As reported in Table 1, the two groups did not significantly differ by age or gender.

Results from Student’s *t*-tests showed that subjects belonging to the BDP-AT group scored significantly higher than BPD subjects, not only in all AdAS Spectrum domains and total score, but also in both MOODS-SR *depression* and *mania* scales as well as in its total, and in all three RRS domains and total score (see Table 2). 

Similarly, further Student’s *t*-tests highlighted not only that BPD-AT subjects scored significantly higher in all MOODS-SR sub-domains scores compared to BPD subjects, but also that they scored higher in MOODS-SR *suicidal ideation* items (see Table 3).

Results from the first logistic regression that had featured the presence of clinically relevant ATs as the dependent variable and MOODS-SR and RRS total scores as independent variables showed that RRS total score was the only significant predictor of the presence of clinically relevant ATs (see Table 4).

Two further logistic regressions were performed using the presence of clinically relevant ATs as the dependent variable and RRS domains in the first (see Table 5) and MOODS-SR domains in the second (see Table 6), as independent variables. Results highlighted the RRS *depression* domain and the MOODS-SR *rhythmicity* domain as significant positive predictors of the presence of clinically relevant ATs.

Results from the last logistic regression performed using the presence of clinically relevant ATs as a dependent variable and MOODS-SR *Suicidal ideation* and *Suicidal behaviors* as independent variables showed that *Suicidal ideation* was a significant positive predictor of the presence of significant ATs (see Table 7).

## 4. Discussion

Our study aimed to evaluate the correlates of autistic traits in a population of BPD patients, in relation to affective symptoms, rumination, and suicidality.

For this purpose, we divided a sample of BPD subjects in two groups based on the presence—or absence—of clinically relevant ATs. No differences in age and gender emerged between groups. As expected, subjects belonging to the BPD-AT group scored significantly higher at the AdAS Spectrum questionnaire as well as in all its domains compared to those in the BPD-only group, confirming the subdivision made between the two groups.

Interestingly, our results reported the BPD-AT group patients also scored significantly higher than BPD-only patients in the total MOODS-SR, in both its depressive and manic subscales, and in each one of its domains. These findings are consistent with the current literature, highlighting not only the high rates of comorbidity between full-blown ASD and mood disorders, with an estimated prevalence of coexisting ASD and mood disorders up to 30% [67,68], but also the even more frequent co-occurrence of ATs and mood disorders among children, adolescents, and adult populations [69,70,71,72,73,74]. In particular, our results are consistent with those of previous studies that highlighted how ATs were found in a mild-to-severe range in 22–46% of subjects diagnosed with major depressive disorder and in 50% of subjects with a diagnosis of bipolar disorder, significantly higher than their matched healthy controls, and also seemed to be positively correlated with the severity of the depressive symptoms [12]. Our results, along with those evidences, support the hypothesis that the presence of significant ATs, especially in vulnerable population such as patients with BPD which show high comorbidity and several overlapping features with mood disorders could be associated with a higher presence of depressive or (hypo)manic symptoms [31,75]. Strengthening this hypothesis, an interesting longitudinal study conducted by Taylor et al. investigated whether the presence of high levels of ATs in childhood could be predisposing to develop hypomanic symptoms in adolescence [76]. Similarly, other specific genetic studies have found an overlap in genetic susceptibility factors between ASD and bipolar disorder [77,78,79,80]. Unfortunately, although promising, data on the topic are still sometimes contradictory, with other papers failing to find such correlation [81,82]. Furthermore, the finding of higher scores on the MOODS subscales that analyze the depressive and manic components are in line with the widely recognized association between ATs and depressive or manic symptoms [83,84,85,86]. For instance, the difficulties in getting involved in significant and satisfactory relationships, together with the limited adaptive skills typical of the autism spectrum, would lead to an increased chance of being isolated, and developing loneliness and consequently a depressive mood, especially during key life transitions [73,87]. Similarly, the lack of empathy and the difficulties in imagination can lead to misunderstanding of the social environment, and consequently, more stressful interpersonal conflicts and challenging situations [88], creating a vicious bidirectional circle. Despite the promising results, some authors have expressed doubts about this correlation, suggesting that subjects with major depression could manifest some symptoms mimicking autistic behaviors (i.e., social withdrawal), leading to confounding results [12], or that the link between AT and depressive symptoms could be related to some psychopathological peculiarities of the subjects [88].

Rumination, intended as negative ruminative thinking, is defined as a recurrent pattern of maladaptive and repetitive thoughts, often linked to anxiety disorders and depression, which can hinder problem-solving skills and the processing of negative emotions and cause social isolation [89,90]. Interestingly, BPD-AT subjects scored significantly higher also in the RRS total and in all its domains compared to the BPD-only group, highlighting in the first group a greater tendency toward rumination. Moreover, from our results emerged that both RRS total score and its depression domain, were significantly predictive of the presence of clinically relevant ATs. This data confirms the evidences already present in the contemporary literature that report how on one hand rumination is a key symptom in ASD [91], and on the other, in a broader perspective, ruminative thinking can be considered as a transnosographic dimension present in several psychiatric disorders, including BPD, associated with a worse prognosis and suicidal thoughts, and possibly underlain by the presence of ATs [92,93,94]. Interestingly, some authors have proposed that rumination plays a major part in BPD symptoms. In particular, rumination has been suggested to be a component of emotional dysregulation in people with BPD, as per the Emotional Cascade Model [95,96]. This process involves an emotion-arousing event that causes negative emotion, which in turn causes rumination, which in turn intensifies the negative emotion [97]. Different types of ruminative thinking content may have different effects on mood and behavior, and the substance of rumination can vary as well. In this context, according to recent studies, ruminative thought is common in people with BPD, with anger rumination being the most common kind [98]. Research has shown that this way of thinking strengthens behavioral discontrol and raises unpleasant feelings [97,98,99]. Moreover, anger rumination has been found to be significantly correlated with the severity of BPD, specifically symptoms including sorrow, anger, and overall negative affect [100]. Similarly, a recent study highlighted how impulsive behaviors, including the tendency toward aggressiveness in individuals with BPD, were predicted by angry rumination [100,101,102].

In this framework, the results of the depression domain being a significant predictor of the presence of ATs may be in line with the above-mentioned literature, suggesting that BPD subjects with more severe depressive ruminative thinking may be characterized by greater ATs. As stated above, while the presence of ATs has been linked to the development of mood disorders and to their severity [12,39,103,104], recent research highlighted not only the persistence of depressive symptoms and depressive ruminations from childhood to adulthood in people with elevated ATs, but also the failure to recognize and treat them when they occur together with ATs themselves [73,105]. It is noteworthy that MOODS rhythmicity domain also emerged as a significant predictor of the presence of relevant ATs. This finding is consistent with the growing evidence about the existence of a correlation between circadian rhythm alterations and ASD [32,33]. It is now widely known that the circadian timing system plays a key role in regulating several physiological functions (i.e., sleep–wake cycles) and cognitive performances (i.e., language, communication and social behaviors) [106,107]. Moreover, biological rhythmic dysfunctions are associated with several human diseases, including psychiatric and neurological conditions [108,109,110]. Previous studies reported that sleep altered patterns are frequent in children with ASD (50–80%) [111], with a higher prevalence with respect to other neurodevelopmental disorders [112]. In details, children with ASD showed several sleep disorders, including prolonged sleep latency, reduction in total sleep duration, atypical REM (rapid eye movements) sleep patterns, and higher rates of REM sleep behavior disorders [112,113,114,115]. Considering these findings, and the evidence that sleep alterations have negative consequences on brain maturation and cognitive functions [116], some authors hypothesized a role of circadian alterations in ASD pathophysiology, while, on the other hand, symptoms of ASD may alter circadian rhythms, creating a vicious bidirectional circle [33]. Nevertheless, if biologic rhythms and environment interact in controlling several physiological pathways, including brain development and behavioral processes, the impaired regulation of the core clock neurodevelopmental signaling could correlate with ASD clinical heterogenic presentation [108,117]. Supporting this finding, sleep altered patterns seemed to be different among ASD patients with different severity of cognitive impairment [118]. In this framework, a recent paper of Lorsung et al. reviewed the link between circadian dysfunctions and ASD [33], highlighting the growing evidence that ASD and ATs populations show abnormal sleep–wake cycles and several alterations in diurnal profiles of typical circadian biomarkers, including melatonin, cortisol, and serotonin levels. Considering epidemiological, genetic, and biochemical studies, the authors suggested some explanations of circadian alterations found in ASD individuals, including birth timing factors, polymorphisms in Clock Genes, and alterations in the mTOR pathway [33]. Considering the link between altered rhythmicity and mood disorder, it is also possible that rhythmicity alterations linked to an autism spectrum may further facilitate the development of mood symptoms in vulnerable subjects as BPD patients [9]. Finally, our results showed how BPD-AT subjects achieved significantly higher scores on suicidal ideation than the BDP only group, and how suicidal ideation itself emerged as a positive predictor of the possible presence of underlying ATs in these subjects.

These data are in line with the current literature confirming the presence of an association between both ASD and subthreshold ATs and an increased risk suicidal ideation [45,119,120,121]. This data is of particular interest considering that patients with BPD very often report suicidal thoughts, suggesting that the presence of ATs may be linked to this particular feature. Among the main factors increasing suicidal risk, difficulties in the social environment and the consequent social isolation that derives from it, the risk of suffering acts of bullying or physical/sexual violence, and the difficulty in adapting to changes in routine, typical of subjects with high ATs, have been suggested to play a central role [119,122,123]. Moreover, the presence of mood disorders and anxiety disorders, more frequent in subjects with ASD as well as in subjects with high ATs, is also known to increase the vulnerability towards both suicidal ideation and behaviors [124,125,126,127]. Our results support previous evidence that reported that a high percentage of suicidal subjects generally obtains report scores indicative for ASD or clinically relevant ATs on autism spectrum-related questionnaires [110,128]. On this basis, many authors have further stressed the importance of delving deeper into this correlation, in order to design a more targeted anti-suicide prevention aimed at subjects on the ASD spectrum, who due to their neuroatypicality may appear to be at greater risk of suicidal tendencies since childhood [110,128].

Our results should be seen in light of some limitations. For instance, the cross-sectional model of our research prevents us from drawing conclusions regarding both temporal and causal connection between the variables. Second, the study was conducted with self-report questionnaires and as a consequence participant over- or underestimation of symptoms could have potentially biased the outcomes of the scores. Furthermore, since the recruitment was carried out on a consecutive basis in patients attending the psychiatric clinic of Pisa, and since patients who presented depressive or anxious symptoms, even if lower than those of BPD, were not excluded, our results could be influenced by sample selection bias. Also, the comments about genetic similarities and/or problems with circadian rhythms should be considered taking into account their speculative nature. Moreover, potential confounding factors such as comorbidity, medication status, or socioeconomic factors, which could influence both AT and mood symptoms, were not investigated. Finally, the limited size of our sample limits the extensibility of our data. Further studies in wider samples, possibly with a longitudinal design, are needed to clarify the implications of the presence of autistic traits among patients with BPD.

## 5. Conclusions

Despite these limitations, our results seem to support the existing, although yet scarce, evidence about the presence of a link between autistic traits and BPD, and contemporarily highlight the presence of autistic traits in this population of specific correlates, which may worsen the clinical picture. In particular, a greater severity of mood symptoms, a higher tendency towards depressive ruminative thinking, and an increased presence of suicidal ideation have been highlighted among BPD subjects with autistic traits.

## Figures and Tables

**Table 1:** Age and gender comparison between diagnostic groups.

		BPD	BPD-AT	t	*p*
		Mean ± SD	Mean ± SD		
**Age**		35.11 ± 9.33	33.31 ± 10.37	0.610	0.545
		***n* (%)**	***n* (%)**	**Chi-Square**	***p***
**Gender**	**F**	12 (63.2%)	21 (72.4%)	0.458	0.499
	**M**	7 (36.8%)	8 (27.6%)		

**Table 2:** Comparison of scores obtained in AdAS Spectrum, MOODS-SR, and RRS questionnaires among groups.

	BPD Mean ± SD	BPD-AT Mean ± SD	t	Cohen’s d	*p*
	AdAS Spectrum scores				
**Child./Adolesc.**	4.53 ± 2.48	8.79 ± 3.74	−4.35	3.321	<0.001 *
**Verb. comm.**	3.42 ± 1.95	7.31 ± 3.30	−5.13 *	2.847	<0.001 *
**Non-verb. comm.**	5.37 ± 2.65	12.65 ± 4.59	−6.96 *	3.948	<0.001 *
**Empathy**	2.21 ± 1.96	4.69 ± 2.80	−3.45	2.508	0.002 *
**Inflex. and routine**	6.79 ± 3.52	16.34 ± 5.63	−6.58	4.916	<0.001 *
**Restrict. Interest and rum.**	4.10 ± 2.86	10.69 ± 3.81	−6.43	3.470	<0.001 *
**Hyper/hyporeact.**	1.42 ± 1.30	6.14 ± 3.69	−6.31 *	2.993	<0.001 *
**Total Score**	27.84 ± 8.89	66.62 ± 19.34	−9.39 *	16.080	<0.001 *
	**MOODS-SR scores**				
**Total Mania**	8.68 ± 6.49	14.93 ± 7.07	−3.09	6.846	0.003 *
**Total Depression**	25.74 ± 8.03	32.83 ± 8.15	−2.97	8.100	0.005 *
**Total Score**	52.58 ± 24.43	79.83 ± 23.61	−3.86	23.938	<0.001 *
	**RSS scores**				
**Reflection**	9.39 ± 2.48	12.27 ± 2.62	−3.75	2.565	0.001 *
**Brooding**	11.22 ± 2.60	14.55 ± 2.86	−4.01	2.766	<0.001 *
**Depression**	26.17 ± 6.64	35.78 ± 7.34	−4.49	7.083	<0.001 *
**Total Score**	46.78 ± 10.86	62.89 ± 11.27	−4.80	11.113	<0.001 *

* Significant for *p* < 0.05.

**Table 3:** Comparison of scores obtained in MOODS-SR questionnaire domains among groups.

	BPD Mean ± SD	BPD-AT Mean ± SD	t	Cohen’s d	*p*
**Depressive Mood**	22.95 ± 6.54	27.76 ± 6.42	−2.52	6.471	0.015 *
**Manic Mood**	6.16 ± 5.00	10.72 ± 5.36	−2.96	5.222	0.005 *
**Depressive Energy**	2.79 ± 2.27	5.07 ± 2.56	−3.15	2.545	0.003 *
**Manic Energy**	2.53 ± 1.71	4.21 ± 2.27	−2.75	2.072	0.009 *
**Depressive Cognition**	7.63 ± 5.38	13.38 ± 5.98	−3.39	5.751	0.001 *
**Manic Cognition**	3.16 ± 3.92	6.45 ± 4.63	−2.55	4.368	0.014 *
**Rhythmicity**	7.37 ± 4.76	12.24 ± 4.07	−3.79	4.353	<0.001 *
	**MOODS-SR Suicidality**				
**Suicidal ideation**	1.50 ± 1.51	2.95 ± 1.27	−2.74		0.011 *
**Suicidal behaviors**	0.80 ± 0.92	1.08 ± 0.86	−0.742		0.466

* Significant for *p* < 0.05.

**Table 4:** Logistic regression analysis with presence of clinically relevant ATs as a dependent variable and MOODS-SR and RRS total as independent variables.

	**b (SE)**	***p***	**Exp (B)**
*constant*	−6.181 (1.931)	0.001 *	0.002
**RRS total score**	**0.121 (0.035)**	0.001 *	**1.129**
**Variables not in the equation**			
	**Score**	***p***	**df**
**MOODS-SR total score**	2.837	0.092	1

Cox and Snell R^2^ = 0.326; Nagelkerke R^2^ = 0.442; * significant for *p* < 0.05; global correct percentage: 76.1%. Variables not in equation.

**Table 5:** Logistic regression analysis with presence of clinically relevant ATs as a dependent variable and RRS domains scores as independent variables.

	**b (SE)**	***p***	**Exp (B)**
*constant*	−5.261 (1.733)	0.002 *	0.005
**RRS Depression**	0.185 (0.056)	0.001 *	1.203
**Variables not in the equation**			
	**Score**	***p***	**df**
**RRS Reflection**	0.355	0.551	1
**RRS Rumination**	1.461	0.227	1

Cox and Snell R^2^ = 0.303; Nagelkerke R^2^ = 0.410; * significant for *p* < 0.05; global correct percentage: 78.3%.

**Table 6:** Logistic regression analysis with presence of clinically relevant ATs as a dependent variable and MOODS-SR domains scores as independent variables.

	**b (SE)**	***p***	**Exp (B)**
*constant*	−1.964 (0.835)	0.019 *	0.140
**MOODS-SR Rhythmicity**	0.241 (0.078)	0.002 *	1.272
**Variables not in the equation**			
	**Score**	***p***	**df**
**Depressive mood**	0.349	0.555	1
**Manic mood**	1.227	0.268	1
**Depressive energy**	1.125	0.289	1
**Manic energy**	0.644	0.422	1
**Depressive cognition**	0.519	0.471	1
**Manic cognition**	1.058	0.304	1

Cox and Snell R^2^ = 0.226; Nagelkerke R^2^ = 0.306; * significant for *p* < 0.05; global correct percentage: 77.1%.

**Table 7:** Logistic regression analysis with presence of clinically relevant ATs as a dependent variable and MOODS-SR suicidality as independent variables.

	**b (SE)**	***p***	**Exp (B)**
*constant*	−1.752 (1.096)	0.110	0.173
**Suicidal ideation**	0.867 (0.389)	0.026 *	2.379
**Variables not in the equation**			
	**Score**	***p***	**df**
**Suicidal behaviors**	0.518	0.472	1

Cox and Snell R^2^ = 0.263; Nagelkerke R^2^ = 0.354; * significant for *p* < 0.05.

## Data Availability

All data generated or analyzed during this study are included in this published article, further inquiries can be directed to the corresponding author.
